# Red blood cell alloimmunisation in multi-transfused patients from an haemodialysis service in Burkina Faso

**DOI:** 10.4102/ajlm.v11i1.1625

**Published:** 2022-09-26

**Authors:** Koumpingnin Nebie, Salam Sawadogo, Salifo Sawadogo, Jérôme Koulidiati, Habi Y.A. Lengani, Abdoul G. Sawadogo, Jérôme Babinet, Mohammed Khalloufi, Saliou Diop, Eléonore Kafando

**Affiliations:** 1 National Blood Center of Ouagadougou, Ouagadougou, Burkina Faso; 2 Laboratory of Haematology, Department of Fundamental Sciences, Health Sciences Research and Training Unit, University Joseph KI-ZERBO, Ouagadougou, Burkina Faso; 3 National Institute for Medical Sciences, University Nazi Boni, Bobo-Dioulasso, Burkina Faso; 4 Souro Sanou Teaching Hospital, Bobo-Dioulasso, Burkina Faso; 5 Yalgado Ouedraogo Teaching Hospital, Ouagadougou, Burkina Faso; 6 Centre National de Référence pour les Groupes Sanguins (CNRGS), National Institute for Blood Transfusion, Paris, France; 7 French Establishment of Blood, Bobigny, France; 8 Department of Haematology, University Cheikh Anta Diop, Dakar, Senegal; 9 Laboratory of Haematology, Paediatric Teaching Hospital Charles de Gaulle, Ouagadougou, Burkina Faso

**Keywords:** blood transfusion, alloimmunisation, RBC antibody, CRF, Burkina Faso

## Abstract

**Background:**

In Burkina Faso, red blood cell (RBC) transfusion remains the crucial anaemia treatment following chronic renal failure (CRF) as erythropoietin and its analogues are unavailable. However, blood group matching beyond the ABO and Rhesus is not common in Burkina Faso. Thus, alloimmunisation is a potential issue for transfused patients.

**Objective:**

Our study aimed to identify anti-erythrocyte antibodies in multi-transfused CRF patients at the Yalgado Ouedraogo Teaching Hospital, Ouagadougou, Burkina Faso.

**Methods:**

This cross-sectional study, conducted from October 2018 to November 2019, included CRF patients who had received at least two RBC units. We screened patients for the presence of RBC antibodies using three commercial Cells panels and identified antibody specificities for positive screenings using 11 Cells panels for an indirect antiglobulin test (IAT) in a low ionic strength microcolumn gel-card system.

**Results:**

Two hundred and thirty-five patients (45.1% female; average age: 41.5 years) were included. The median number of blood units received per patient was 10 (interquartile range: 5–20). The overall alloimmunisation rate was 5.9% (14/235). Antibodies identified included: anti-D (1 case), anti-C (1 case), anti-D+C (4 cases), anti-C^W^ (1 case), anti-E (1 case), anti-S (1 case) and anti-Le^a^ (1 case). In four positive patients, the specificity of the antibodies was indeterminate. No risk factors were associated with alloimmunisation.

**Conclusion:**

In Burkina Faso, screening for RBC alloantibodies should be mandated for patients at risk. The high rate of indeterminate antibodies suggests the need to develop a local RBC antibody panel adapted to the local population.

## Introduction

Blood transfusion, specifically the transfusion of red blood cells (RBC), significantly contributes to the modern healthcare system. Every day, blood transfusion saves lives in developing countries where acute anaemia caused by malaria, sickle cell disease (SCD), pregnancy-related events and other trauma remains high or is on the rise. For example, in Burkina Faso, around 103 731 RBC units were used in 2017.^1^ However, this did not meet the transfusion needs of the country. Moreover, this number is far lower than the theoretical needs of around 196 000–580 000 per the World Health Organization estimation method III (i.e. 1% – 3% of the 19.5 million inhabitants).^2^

Besides the chronic blood shortage, developing countries also face poor quality of blood products and their unsafe use.^3^ Indeed, residual risks of transfusion-transmitted infections remain high^4,5^ due to inadequacies in blood donor selection and retention and laboratory screening of blood donations. Furthermore, blood transfusion adverse events are underestimated due to the weakness or nonexistence of haemovigilance and quality management systems.^6,7^ Finally, although transfusion-transmitted infections and major blood groups matching errors are worrying, blood transfusion safety issues, alloimmunisation and the occurrence of alloantibody are also pressing issues, especially in multi-transfused patients such as those undergoing haemodialysis for chronic renal failure (CRF).^4,8,9,10^

Anaemia is highly prevalent in end-stage renal disease patients, often non-regenerative normochromic normocytic anaemia caused by inadequate renal erythropoietin production. Erythropoietin infusions or other erythropoietin-stimulating agents manage anaemia in end-stage renal disease patients. The United States Food and Drug Administration recommends an erythropoietin haemoglobin target range of 100 g/L – 120 g/L^11^ and expressly states that erythropoietin-stimulating agents should be used to increase haemoglobin only to the level necessary to avoid transfusion.^11,12,13^ In 2016, an expert committee advocated for including erythropoietin-stimulating agents in the World Health Organization Model List of Essential Medicines to reduce the need for transfusions in patients with end-stage chronic kidney disease. Erythropoietin-stimulating agents prevent transfusion-related risks, facility requirements, and risk management costs in the event of possible harm (infections, haemosiderosis).^14^

However, erythropoietin-stimulating agents treatments are out of reach for most patients in our context. Therefore, RBC transfusion is used to manage CRF-related anaemia and SCD patients. Meanwhile, our country faces poor pre-transfusion compatibility practices; ABO and RhD matching is the only mandatory screening for RBC transfusions. No other blood group is considered, and no alloantibody screening or compatibility test is performed.^15^ Given this context, high RBC alloantibodies frequency is expected among transfused patients; however, there is a paucity of data on this. Thus, this study determined the frequency of anti-erythrocyte alloimmunisation and identified alloantibody specificities among CRF multi-transfused patients in Yalgado Ouedraogo Teaching Hospital, Ouagadougou, Burkina Faso.

## Methods

### Ethical considerations

Both the Yalgado Ouedraogo Teaching Hospital direction and the internal ethical committee of the national blood transfusion centre (Authorisation no. 015/CNTS/DG/CIRS, 03/23/2018) approved the study. The nurses and the medical doctor in charge of the interview and other data collection obtained verbal informed consent. Also, the data were password protected and accessible only by the first author. Results were shared with staff and patients and used to influence patients’ future transfusions.

### Study setting

This study was conducted in the nephrology and haemodialysis unit of the teaching hospital Yalgado Ouedraogo of Ouagadougou, Burkina Faso, where about 400 patients with chronic kidney failure undergo haemodialysis yearly.

We conducted this cross-sectional study from January 2018 to December 2019 and included haemodialysis end-stage chronic kidney failure patients who had ever received RBC transfusions at least twice. Socio-demographic information, clinical data, and medical history of each included patient were recorded on a standardised survey form during an in-person interview with the medical doctor responsible for the study or trained nurses. Data collected include gender, age, date of the first transfusion, date of the last transfusion received, number of transfusions, the total number of blood units received since CRF started, and number and type of adverse reactions related to transfusions reported. Additionally, the number of pregnancies, live and still births, abortions, and anti-D injection use were also reported for female patients. Five mililitres of blood was drawn from each patient into ethylenediaminetetraacetic acid tubes. The sample was centrifuged, and the obtained plasma was used for alloantibodies screening and identification.

### Testing methods

We used the indirect antiglobulin test method with the gel column agglutination card technique (Invitrogel AHG, MTC Invitro Diagnostics AG, Bensheim, Germany). In this technique, the gel column contains an anti-human antibody that traps irregular antibodies present in a patient’s plasma and fixed on RBC. Agglutinated RBCs are trapped in the gel column, making the agglutination easy to read.^16,17^

In the first step, a panel of three RBC reagents (Invitrocell Screen I-II-II, MTC Invitro Diagnostics AG, Bensheim, Germany), that targets antigens D, C, c, E, e, V, C^W^, K, k, Kp^a^, Kp^b^, Js^a^, Js^b^, Fy^a^, Fy^b^, Jk^a^, Jk^b^, Le^a^, Le^b^, P^1^, M, N, S, s, Lu^a^, Lu^b^ and Xg*a antibodies, were used for screening. Samples positive for any antibody were further tested to identify antibody specificity using an 11 RBC panel (Invitrocell Ident 11, MTC Invitro Diagnostics AG, Bensheim, Germany) that targets the same antigens in the screening stage. An enzyme-treated RBC panel was not used.

### Statistical analyses

We used EPI-INFO^TM^ software (version 7.2.2.2, Centers for Disease Control and Prevention, Atlanta, Georgia, United States) for data analysis. The frequencies and percentages are given with a 95% confidence interval. We used the chi-square test to compare proportions, and differences were considered significant for *p* < 0.05.

## Results

### Baseline characteristics

During the study period, 235 patients with CRF were included, comprising 45.1% (106/235) female patients. The mean age was 41.9 (standard deviation 14.5 years; median 41 years; range 15–86 years). The mean number of received RBC units was 18 units ranging from two to 160 RBC, while the median number of received blood units per patient was 10 (interquartile range: 5–20). About 55.2% (128/232) had received more than 10 RBC units (Table 1).

**TABLE 1:** Social and demographic characteristics of patients with chronic renal failure, Yalgado Ouedraogo Teaching Hospital of Ouagadougou, Burkina Faso, 2018.

Characteristics	Total number of patients	Number of subject in each group	Frequency (%)
**Age (years)**	235		100.0
< 20		8	3.4
20–29		46	19.6
30–39		55	23.4
40–49		47	20.0
50–59		47	20.0
≥ 60		32	13.6
**Gender**	235		100.0
Male		129	54.9
Female		106	45.1
**Number of pregnancies**	106		100.0
0–1		27	26.7
At least 2		74	73.3
**Number of red blood cell units received**	231		100.0
2–9		103	44.6
10–19		65	28.1
20–29		32	13.9
At least 30		31	13.4
**Duration from last transfusion**	228		100.0
Less than 3 months		120	52.6
More than 3 months		108	47.4
**ABO blood group**	235		100.0
AB		11	4.7
A		72	30.6
B		50	21.2
O		102	43.5
**Rhesus D**	235		100.0
Negative		9	3.8
Positive		226	96.2
**History of adverse events**	218		100.0
Present		71	32.6
Absent		147	67.4

### Red blood cell alloimmunisation prevalence

Of the 235 patients included, 14 had alloantibodies, representing an overall positivity rate of 5.9%. Four of the 14 patients (28.6%) had indeterminate antibody specificity. In 10 patients, 14 antibodies were identified: 5 anti-D, 5 anti-C, 1 anti-E, 1 anti-C^w^, 1 anti S, 1 anti-Le^a^; four patients were positive for both anti-D and anti-C (Table 2).

**TABLE 2:** Characteristics of patients with red blood cell alloimmunisation, Yalgado Ouedraogo Teaching Hospital of Ouagadougou, Burkina Faso, 2018.†

Patient ID	Antibodies specificity	Gender	Age (years)	Number of red blood cell units received	Number of pregnancies
76	Anti-D + -C	Female	20	20	0
101	Anti-C^w^	Female	29	12	3
119	Anti-S	Female	56	12	8
173	Anti-C	Female	49	21	3
178	Anti Le^a^	Female	52	8	4
185	Anti-D + -C	Male	33	20	-
199	Anti-E	Female	70	1	6
235	Anti-D + -C	Female	21	15	0
255	Anti-D + -C	Male	30	10	-
262	Anti-D	Male	73	11	-
128	Undetermined	Male	38	35	-
213	Undetermined	Male	61	6	-
225	Undetermined	Male	60	55	-
238	Undetermined	Male	45	15	-

† , This analysis included 14 of the 235 patients with chronic renal failure included in the study.

Most antibodies (12 of 14; 85.7%) were of the anti-RH blood group antigens, with anti-D and anti-C being the most prevalent, each accounting for 35.7% (Table 3).

**TABLE 3:** Specificity and frequency of alloantibodies found among multi-transfused patients of the Yalgado Ouedraogo Teaching Hospital of Ouagadougou, Burkina Faso, 2018.†

Type of Antibody	Number	Frequency (%)
Anti-D	5	2.1
Anti-C	5	2.1
Anti-E	1	0.4
Anti-c	-	-
Anti-e	-	-
Anti-C^w^	1	0.4
Anti-S	1	0.4
Anti-Le^a^	1	0.4
Undetermined	4	1.7
All	14	5.9

† , This analysis included all 235 patients with chronic renal failure included in the study.

### Red blood cell alloimmunisation risk factors

There were no differences in the mean age (43.3 vs 41.8 years, *p* = 0.21) and the mean number of blood units received (13.0 vs 15.7 RBC units, *p* = 0.36) between immunised and non-immunised patients. The alloimmunisation rate was higher in patients who had received more than 10 RBC units (7.4% vs 2.3%, *p* = 0.12), but this difference was statistically insignificant. There were no other factors associated with alloantibodies (Table 4).

**TABLE 4:** Factors associated with alloimmunisation in multi-transfused patients with chronic renal failure, Yalgado Ouedraogo Teaching Hospital, Ouagadougou, Burkina Faso, 2018.

Characteristic	Number of patients†	Positive patients	Percentage (%)	*p*	Statistical significance
**Age (years)**	231			0.62	Not significant
≤ 30	65	4	3.6		
> 30	166	6	6.1		
**Gender**	231			0.21	Not significant
Female	106	7	6.6		
Male	125	3	2.4		
**Pregnancies** ‡	101			1.00	Not significant
0–1	27	2	7.4		
≥ 2	74	5	6.8		
**Red blood cell units received**	228			0.12	Not significant
≤ 10	133	3	2.3		
> 10	95	7	7.4		
**Duration from last transfusion (months)**	226			0.28	Not significant
≤ 3	120	6	5.0		
> 3	106	2	1.9		

† , Total numbers for these group is < 235 due to missing data;

‡ , This analysis included only adult female patients.

## Discussion

Our study aimed at determining the frequency and the specificity of alloantibodies among the multi-transfused haemodialysis CRF patients at the teaching hospital Yalgado Ouedraogo of Ouagadougou (Burkina Faso). We found an alloimmunisation rate of 5.9% with antibodies mainly of the anti-Rh blood group antigens specificity.

This study overviews of RBC immunological risks among patients with chronic diseases who are lifelong blood transfusion patients. Although there have been recent changes in the blood transfusion system in Burkina Faso, including the replacement of multiple hospital-based blood banks with a centralised system, standardisation and harmonisation of practices,^6,18,19,20^ improved blood collection and infectious disease screening, some improvements towards managing blood recipients are necessary. For example, compactibility screening is still limited to the ABO and RhD antigens, contrary to obtainable standards in high-income countries, where rare groups, at least Rh-Kell major antigens, are screened for before transfusion. Moreover, in Burkina Faso, alloantibody screening tests and laboratory compatibility tests using at least an indirect antibody test as recommended is not implemented: the patient’s plasma and a sample of the RBC units are tested for agglutination on a glass surface.

This study was the first in the country to use the gel column card method, one of the current best methods for alloantibody screening. Nevertheless, the study presents some limitations as complementary antibody identification techniques, such as enzyme-treated red cells reagents panels (papain, bromelain or other) or wide-range panels, were not used. The lack of complementary identification can explain the high rate (4 of 14; 28.7%) of alloantibody undetermined specificity (inconclusive antibody identification).^21^

The overall alloimmunisation rate of 5.9% among CRF patients undergoing haemodialysis on our study is consistent with the findings of two systematic reviews and meta-analysis studies conducted by Ngoma et al. in 2015 and Boateng et al. in 2019. These studies reported an overall alloimmunisation rate of 6.95% and 7.5% in sub-Saharan Africa.^22,23^ Our results are similar to those of Kafando et al., who found an alloimmunisation rate of 4.2% among children transfused with Rh-Kell unmatched blood units.^24^ Alloimmunisation rates in the same range were reported in Uganda (6.1%), Rwanda (6.4%), Sudan (4.0%) and Tanzania (4.1%). However, some reported higher rates: Uganda in 2010 (10.2%), Mali in 2013 (10.3%) and Nigeria in 2015 (9.3%).^22,25,26,27,28^ These results reflect the poor immunological safety of blood transfusions in sub-Saharan Africa, where blood transfusion is performed based only on the blood donor and recipient ABO and RhD antigens matching.

Alloimmunisation rates observed in our study and other studies from sub-Saharan Africa are lower than those observed in Europe and North America when they only screened for ABO and RhD. Prevalences ranged from 18% to 76% in the United Kingdom and United States.^29,30,31,32,33,34^ In France, the rate was about 30%.^35,36^ Despite the mandatory donor and recipient Rh-Kell antigens matching before transfusion in these developed countries, alloimmunisation rates in those settings are higher than ours.^34,37,38^ This serves as a reminder that the risk of alloimmunisation is multiparametric, depending on the population’s subgrouping or prevalent diseases.^30,36^ Also, high rates of alloimmunisation could be due to antigen discrepancies between transfused RBC concentrates collected from donors with European ancestry and SCD recipients who are often of sub-Saharan African descent.^36,37^ A similar hypothesis was assumed in some other countries with multi-ethnic groups, such as Iran.^39^

In Burkina Faso, blood group antigen distribution is established for ABO and RhD within blood donors and patients.^40,41^ There is no data about Rh subgroups or other important RBC antigens. It is known that significant differences in the distribution of blood group antigens within the country’s natural ethnic groups could exist. Sawadogo et al. found that the phenotype O was more frequent in the Central-West, Central and East regions corresponding to ‘Mossi’, ‘Gourounsi’, and ‘Gourmantché’ areas, whereas the phenotype A and AB were more prevalent in ‘Boucle du Mouhoun’ and ‘Hauts-Bassins’ regions and the ‘Bwaba’ and ‘Bobo’ areas. The phenotype O negative was infrequent in ‘Bwaba’.^40^ These studies suggest that in Burkina Faso, with more than 50 ethnic groups, dominant blood groups vary between or are specific to particular ethnic groups. Thus, new studies should be conducted to establish blood subgroup frequencies and RBC matching strategies in the country.

In our study, the antibody specificity of four participants of 14 (28.6%) was indeterminate. This impairment could be due to the discrepancy between the European-sourced red cell reagent panel and our population. This situation highlights the need to implement local panels for RBC alloantibodies testing as with some other low- and middle-income countries.^42,43,44^ Furthermore, Boateng et al. claim that creating and maintaining a database of phenotyped blood donors will facilitate the selection of matched blood components for emergency transfusions as seen in sub-Saharan Africa and help locally manufacture RBC reagents. Thus, RBC alloantibodies screening may become more economical and sustainable for multi-transfused patients, particularly patients with SCD in this zone.^22^

The majority (85.7%) of the alloantibodies found in this study were anti-Rh group antigens. Anti-D and anti-C antibodies accounted for 35.7%, followed by anti-E and anti-C^w^. In a previous study of children who received transfusions in Burkina Faso, anti-C and anti-E were the most frequent.^24^ In our study, the two mainly represented antibodies were co-associated (Anti-D+C) in 4 of 14 patients. The predominance of Rh group antibodies was also reported in some other west African countries, as well as in Côte d’Ivoire,^45^ Mali,^26^ Senegal^46^ and Nigeria,^27,47^ but in these studies, anti-E was the most often encountered when compared to anti-D and anti-C. Surprisingly, we found Rh anti-D antibodies, which could be due to errors occurring during patients’ blood typing. Our hospital has reported as many as 46 blood typing errors yearly (unpublished data). Another hypothesis is that partial RhD antigen carriage is frequent in individuals with African ancestry. In this case, an RhD-positive patient can develop alloantibodies after receiving RhD-positive RBC, as reported by Chou et al.^30,48^ The same hypothesis applies to partial C carriers.^49^

This study tried to identify the risk factors associated with alloimmunisation. Neither gender, age, nor the number of blood units received was associated with alloimmunisation in our study. However, Ifeoma et al.^47^ in Nigeria, Senghor et al.^46^ in Senegal and Natukunda et al.^50^ in Uganda have associated these factors with alloimmunisation; the small size of our sample might have prevented the observation of such associations.

### Limitations

One limitation of this study was that we could not screen for antibodies within a reasonable time after each transfusion. For many patients, screening occurred months or years after the last transfusion event. This delay may have impacted the alloimmunisation rate.

### Conclusion

This study showed that RBC alloimmunisation is a reality in multi-transfused patients in Burkina Faso. Therefore, exhaustive donor-patient blood matching beyond ABO and RhD is necessary for lifelong transfused patients, such as CRF and SCD patients. Further investigations are needed to efficiently establish the distribution of RBC antigens and phenotypes among blood donors and patients in the country, which may facilitate RBC reagent manufacturing.
